# Techniques of staging laparoscopy and peritoneal fluid assessment in gastric cancer: a systematic review

**DOI:** 10.1097/JS9.0000000000000632

**Published:** 2023-08-14

**Authors:** Karol Rawicz-Pruszyński, Maria Erodotou, Zuzanna Pelc, Katarzyna Sędłak, Wojciech Polkowski, Timothy M. Pawlik, Bas P.L. Wijnhoven

**Affiliations:** a Department of Surgical Oncology, Medical University of Lublin, Lublin, Poland; b Department of Surgery, The Ohio State University Wexner Medical Center and James Comprehensive Cancer Center, Columbus, Ohio, USA; c Department of Surgery, Erasmus Medical Center, Rotterdam, The Netherlands

**Keywords:** gastric cancer, peritoneal fluid assessment, staging laparoscopy

## Abstract

**Background:**

Staging laparoscopy for gastric cancer is recommended to assess the tumor’s locoregional extension and exclude peritoneal disease. As there is no consensus on optimizing the procedure’s diagnostic accuracy, we aimed to systematically review the literature on operative techniques, followed by peritoneal lavage fluid assessment in gastric cancer patients. Specifically, we sought to indicate the most common characteristics of the procedure and cytological evaluation.

**Methods:**

This study was conducted according to the Preferred Reporting Items for Systematic Reviews and Meta-Analyses (PRISMA). The protocol for this systematic review was registered on PROSPERO database (CRD: 42022306746). On September 2022, a search was carried out using Embase, Medline ALL, Cochrane Central Register of Controlled Trials, and Web of Science Core Collection.

**Results:**

The search identified 1632 studies on staging laparoscopy and 2190 studies on peritoneal fluid assessment. Some 212 studies were included. Open Hasson was the method of choice in accessing the peritoneal cavity in 65% of the studies, followed by establishing a pneumoperitoneum at 10–12 mmHg in 52% of reports. Most frequently, the patient was positioned supine (70%), while a 30° scope and three ports were used to assess the peritoneal cavity clockwise (72%, 77%, and 85%, respectively). Right and left upper abdomen quadrants were the predominant area of laparoscopic exploration (both 65%), followed by the primary tumor region (54%), liver and pelvis (both 30%), and small bowel and spleen (19% and 17%, respectively). Regions of peritoneal lavage and aspiration were limited to the pelvis (50%), followed by right and left upper abdomen quadrants (37.5% and 50%, respectively). No studies compared different methods of operative techniques or analysis of ascites/fluid.

**Conclusions:**

This study indicates a high heterogeneity in the technique of staging laparoscopy and peritoneal fluid assessment in gastric cancer patients. Further research and initiatives to reach a consensus on the standardization of the procedure are warranted.

## Introduction

Highlights

- This systematic review is first to summarize the literature on techniques of staging laparoscopy and peritoneal fluid assessment for gastric cancer.
- The search identified 1632 studies on staging laparoscopy and 2190 studies on peritoneal fluid assessment. In total, 212 studies were included.
- This study indicates a high heterogeneity in the technique of staging laparoscopy and peritoneal fluid assessment in gastric cancer patients. Further research and initiatives to reach a consensus on the standardization of the procedure are warranted.

Gastric cancer is the fifth most common malignancy worldwide, with an annual incidence of 1 000 000 cases^1^. Approximately 50% of patients present distant metastases at diagnosis, with the peritoneum being the most common site of dissemination^2,3^. Cytoreductive surgery with hyperthermic intraperitoneal chemotherapy may improve the prognosis of patients with peritoneal metastases, but the evidence is merely based on non-randomized cohort studies^4^.

European Society of Medical Oncology (ESMO) and National Comprehensive Cancer Network (NCCN) gastric cancer guidelines recommend computed tomography (CT) for clinical staging and risk assessment^5,6^. Nonetheless, CT sensitivity to detect peritoneal carcinomatosis is highly variable, ranging from 23% to 76%^7^. Positron emission tomography (PET)–CT imaging may improve sensitivity to nearly 80%; however, the negative predictive value (NPV) remains as low as 60%^8^. Of note, the accuracy of magnetic resonance imaging (MRI) in diagnosing peritoneal metastases may reach up to 83%^9^, but mainly among patients with severe carcinomatosis. Regardless of imaging choice, the precise assessment of dissemination is often underestimated due to decreased sensitivity for subcentimeter lesions^10^.

In order to improve the evaluation of radiologically and macroscopically occult peritoneal metastatic disease, staging laparoscopy is recommended for every gastric cancer patient with stage cT1b and higher^3,11–13^. Apart from the omission of unnecessary laparotomy in up to 25% of cases, staging laparoscopy enables the assessment of intraperitoneal lavage washings^3^. Positive cytology without macroscopic dissemination (P0Cyt+) is classified as stage IV gastric cancer with poor survival outcomes^14–16^. Although gastrectomy is ineffective in improving survival among this specific group of patients^17^, positive-to-negative cytology conversion after neoadjuvant chemotherapy is a significant prognostic factor, justifying surgical treatment in well-selected cases^18^. However, the cytological analysis lacks standardization, resulting in free cancer cell detection with sensitivity varying from 26 to 70.8%^16,19^.

Given the clinical importance of diagnosing irresectable and incurable disease prior to curative-intent treatment, the current study aimed to systematically review surgical techniques of staging laparoscopy and cytological assessment of peritoneal lavage fluid in gastric cancer patients. Specifically, we sought to evaluate the access, usage of instruments, areas of peritoneal cavity exploration, and potential complications after the procedure, together with assessing clinical considerations of intraperitoneal lavage washings.

## Methods

The following study was conducted according to the Preferred Reporting Items for Systematic Reviews and Meta-Analyses (PRISMA, Supplemental Digital Content 1, http://links.lww.com/JS9/A854, Supplemental Digital Content 2, http://links.lww.com/JS9/A855, Supplemental Digital Content 3, http://links.lww.com/JS9/A856)^20^. The protocol for this systematic review was registered on PROSPERO database (CRD: 42022306746)^21^.

### Search strategy

On September 2022, a search was carried out using Embase (from 1971), Medline ALL (from 1946), and Cochrane Central Register of Controlled Trials (from 1992) for studies on staging laparoscopy for gastric cancer. At the same time, a second search was conducted using the same database with the addition of the Web of Science Core Collection (from 1975) to assess peritoneal fluid in patients with gastric cancer. The search terms included multiple combinations and synonyms of the keywords “gastric cancer”, “gastroesophageal cancer”, “cancer staging”, “laparoscopy”, “peritoneal lavage fluid”, “ascites”, “assessment”, and “cytology”. The complete search strategy is available in the supplementary material (Supplementary Material Tables 1 and 2, Supplemental Digital Content 4, http://links.lww.com/JS9/A857). All duplicate records were removed before the screening.

### Study questions

Two main clinical questions were addressed:

1. Surgical considerations: patient positioning, type of instruments used, peritoneal cavity access, number of ports/trocars, technical details of locoregional tumor assessment, and peritoneal metastases evaluation.
2. Peritoneal lavage considerations: type and volume of fluid used in assessment, the time interval between washings and cytological evaluation, area of fluid aspiration, and comparison of techniques and biomarkers used in the cytological workup.

### Screening and study selection

Two independent authors (K.R.-P. and M.E.) screened the titles and abstracts to identify citations for inclusion. Any reviewer conflicts were resolved by discussion with the senior author (B.P.L.W.). The studies of interest included patients with gastric or esophagogastric cancer who underwent staging laparoscopy or/and peritoneal washings. Systematic reviews, meta-analyses, reviews, editorials/letters, case reports, posters, conferences, animal models, and studies published in languages other than English were excluded. The study selection procedure for the technique of staging laparoscopy and the peritoneal fluid assessment is presented according to the Preferred Reporting Items for Systematic Reviews and Meta-Analyses (PRISMA, Supplemental Digital Content 1, http://links.lww.com/JS9/A854, Supplemental Digital Content 2, http://links.lww.com/JS9/A855, Supplemental Digital Content 3, http://links.lww.com/JS9/A856)^20^ flowcharts in Supplementary Figure 1 (Supplemental Digital Content 5, http://links.lww.com/JS9/A858) and Supplementary Figure 2 (Supplemental Digital Content 5, http://links.lww.com/JS9/A858), respectively.

### Data extraction and data synthesis

Two authors (K.R.-P. and M.E.) extracted data from the included studies, focusing initially on the first author’s name, year of publication, country, cancer type, number of patients, and study period. Next, technical details on the surgical procedure were assessed, including patient positioning(s), peritoneum access, pressure of pneumoperitoneum, number of trocars used, type of laparoscope, regions of exploration, and classification of peritoneal dissemination. Furthermore, aspiration of ascites/peritoneal lavage fluid was evaluated, including timing, the volume of fluid aspirate and volume for cytology, possible type fluid containers used, followed by assessment methods. Finally, additional screening for procedure complications was conducted. The methodological quality of the studies was assessed using the Strengthening the Reporting of Observational Studies in Epidemiology (STROBE) statement^22,23^ and AMSTAR guidelines, Supplemental Digital Content 6, http://links.lww.com/JS9/A859 (methodological quality – high, score 10)^24^. Due to substantial heterogeneity between the studies, a meta-analysis was waived upon the senior author’s discretion and approval. However, a descriptive analysis of all studies on the technical aspects of staging laparoscopy and peritoneal fluid assessment was performed.

## Results

### Characteristics of included studies

The initial literature search identified 1632 studies describing staging laparoscopy technique and 2190 studies reporting peritoneal fluid assessment. After duplicates were removed, 1132 and 1771 studies remained for screening. Based on the titles and abstracts, another 1855 were excluded. The full text was available for 100 studies on staging laparoscopy technique and 130 studies on peritoneal fluid assessment. In total, 212 publications were included in this systematic review. Out of 212 included studies, 175 (83%) were reported, according to the STROBE statement. Eighty-four studies reporting on staging laparoscopy technique were published between 1984 and 2021, with 5316 patients in total^2,3,11–14,19,25–101^. One hundred twenty-eight studies reporting peritoneal fluid assessment were published between 1993 and 2022, including 20 115 patients^11,13,14,18,25,33,34,36,37,40–44,46,49,54,57,59,61,67,70,73,79,85,93,94,98,102–200^. Peritoneal lavage fluid assessment was most commonly assessed with reverse transcriptase-polymerase chain reaction (RT-PCR) (31%), Papanicolau-alone (30.5%), or in combination with Giemsa staining (10%). Molecular biological techniques for detecting cancer were performed in 3% of included studies, whereas 25.5% of the studies did not report information on cytological examination. Nearly half (47.5%) of the studies lacked data on the volume of fluid used for analysis. Specific characteristics of staging laparoscopy technique and peritoneal fluid assessment are shown in Tables 1, 2, respectively.

**Table 1:** Studies included in the technique of staging laparoscopy assessment.

Number of included studies (references)	Year of publication	Median number of patients (IQR)	Continent/country of the study	Access to peritoneal cavity	Number of trocars	Type of laparoscope	Reported regions assessed during laparoscopy	Complications
84 ^2,3,11–14,19,23–35,37–72,77–89,93–99,101,102,136–140^	1984–2021	5316 (127; 40–167)	Europe *(43.5%)*	Open Hasson (55%)	3 (82%)	30° (90%)	Stomach (49%)	Intestinal perforation (5%)
			Japan *(20%)*	Verres (45%)	2 (13%)	0° (5%)	Local tumor extension (63%)	Diaphragmatic perforation (3.5%)
			China *(11%)*		4-5 (5%)	45° (5%)	Peritoneal surface (76%)	Pulmonary infection (2.5%)
			USA *(10%)*			Greater omentum (58%)	Blood loss (2.5%)	
			Other *(15.5%)*			Liver (69%)		
						Diaphragm (44%)		
							Small bowel with mesenterium (25%)	
							Pelvis (35%)	
							Other (30%)	

Other: esophageal hiatus, pancreas, Winslow foramen, celiac trunk, and large bowel.

**Table 2:** Studies included in peritoneal lavage fluid assessment.

Number of included studies (references)	Year of publication	Median number of patients (IQR)	Country	Type of procedure	Volume of sample (ml)	Technique of cytological examination	Biomarker	Sensitivity	Specificity	Accuracy
128 ^11,13,14,18,25,33,36,37,40,41,44,46,49,57,59,61,62,70,73–77,81,86,88,90–92,100,118,120–130,141–222^	1993–2021	20 115 (114.5; 50.5–165)	Japan *(41%)*	Laparoscopy *(55%)*	100 *(20%)*	Papanicolau *(30.5%)*	CEA *(23%)*	*31–88.5%*	*79–97%*	*64–82.5%*
			China *(14%)*	Laparotomy *(33%)*	50 *(8%)*	RT-PCR *(31%)*	FCC *(18%)*			
			Europe *(21%)*	Paracentesis *(12%)*	200 *(8.5%)*	Papanicolau+Giemsa *(10%)*	Other *(15%)*			
			U.S. *(10.5%)*		250-300 *(5.5%)*	Other *(3%)*	No data *(44%)*			
			Korea *(5.5%)*		500 *(4%)*	No data *(25.5%)*				
			Other *(8%)*		1000 *(6.5%)*					
					Unknown *(47.5%)*					

CEA, carcinoembryonic antigen; FCC, free cancer cells; RT-PCR, reverse transcription polymerase chain reaction.

### Staging laparoscopy technique

#### Access and pneumoperitoneum

**Figure 1:**
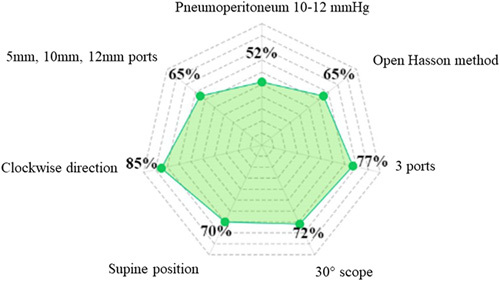
Radar chart indicating the most preferred approach to staging laparoscopy technique among included studies.

The most preferred approach to staging laparoscopy technique among the included studies is depicted in Figure 1. Most studies (17/26, 65.3%) reported using the open Hasson technique for access to the peritoneal cavity^13,32,39,47,48,51,61,63,65,68,70,72,79,84,87,88^. In contrast, in seven (26.9%) studies, the Verres needle was used^39,51,52,64,71,91,94^, while in two studies (7.6%), both methods were proposed^39,51^. The pressure of pneumoperitoneum with CO_2_ was set at 8–15 mmHg, with 12 mmHg being the most commonly reported pressure (4 out of 17 studies, 23%)^47,51,71,94^.

#### Ports/trocars

The number of ports used varied from 1 to 5, as was reported in 57 studies^11–14,19,25,30,32–42,44,45,47–51,53–55,58,59,62,64–68,70–73,75,79–83,85–87,94,96,99,106,142,153^. The optical port was most often placed periumbilical. The maneuver trocar diameter ranged from 5 to 12 mm. The right and left upper quadrants were the most common location for the two remaining trocars. One study reported on a single port Endocam with a 5 mm side channel, which was used for inserting laparoscopic instruments such as biopsy forceps and aspiration of ascites using a urethral stent^72^.

#### Type of laparoscope

A 30-degree laparoscope was predominantly used (27/37 studies, 73%)^11,12,19,30,36–38,47,49,59,64,67,70,81,83,87,94,106,153^.

#### Patient positioning

Most studies reported supine patient positioning (17/23 studies 74%)^12,32–34,40,41,47,49,51,53,55,59,61,67,71,84,94^. In other studies, the patient was in a French or lithotomy position (6/23 studies, 26%)^19,25,30,33,37,94^. Trendelenburg position was reported explicitly in half of the studies (13/26) to visualize the pouch of Douglas, the root of the mesentery, pelvis, and ovaries^47,48,52,61,64,65,68,70,79,84,88,91,94^. Five reports mentioned the anti-Trendelenburg position for better visualization of the upper abdomen by elevating the left liver lobe to approach the anterior surface of the stomach^61,63,70,71,84^. Such maneuvers allowed a more precise assessment of the extent of tumor infiltration on the gastric wall, the perigastric nodes along the greater and lesser curvature, and the gastrohepatic and hepatoduodenal ligaments.

#### Orientation

Seven studies reported on a clockwise exploration of the abdominal cavity to detect peritoneal disease, ascites, liver metastases, or suspected lymph nodes^11,12,25,30,47,51,57^. The inspection started at the right upper quadrant, followed by a visual exploration of the bilateral diaphragmatic dome, the left side of the anterior abdominal wall, the hypogastrium, and the right side of the anterior abdominal wall. A retrospective observational study from China presented a ‘Four-Step Procedure’ in which the examination of the surface of abdominal viscera was according to a so-called ‘S’ route^11^. The exploratory sequence began at the diaphragmatic surface of the left liver lobe, followed by the diaphragmatic surface of the right liver lobe, the surface of transverse colon, the great omentum from left to right, the left side of the abdominal wall, the left paracolic sulcus and the surface of descending colon, the inferior abdominal wall, the surface of the small intestine, the right side of abdominal wall, the right paracolic sulcus, and the surface of ascending colon.

#### Regions of intraoperative assessment

**Figure 2:**
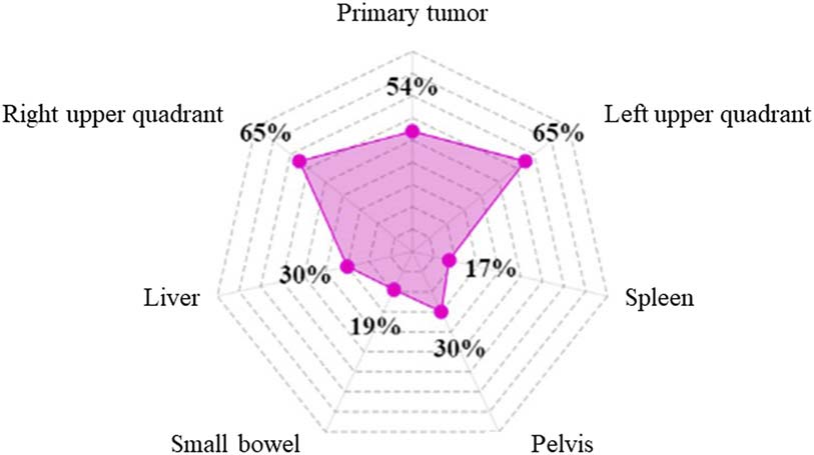
Radar chart evaluating the most common areas of abdomen exploration during staging laparoscopy among included studies.

The most common areas of abdomen exploration during staging laparoscopy among included studies are depicted in Figure 2. Assessment of the primary tumor area and peritoneal cavity was described in detail in 71 studies^11–14,19,25,27,30,32–45,47–51,53–59,61,62,64–75,79–83,85–87,92–96,98–100,106,110,114,142,153,159^. The stomach and assessment of local tumor extension were reported in 35 (49.2%) of the studies, followed by the peritoneal surface (47/71 studies, 65%), pelvis, and Douglas pouch (29/71 studies 30%). Some studies reported specific details of surgical maneuvers to assess local ingrowth of the tumor or sites of peritoneal metastases^19,33,37,57,64,75,85,93,96,110^. These included lifting the left liver lobe to evaluate the complete anterior wall of the stomach, the lesser curvature, the lesser omentum, the undersurface of the left lobe, and hepatoduodenal ligament. In patients with a tumor located at the posterior wall, some studies reported inspection of the lesser sac through a small incision in the gastrocolic ligament or via the opening of the gastrohepatic ligament^12,13,39,41,57,61,64^. To this extent, the relationship of the tumor to retroperitoneal structures such as pancreas, celiac axis, and the peritoneal surface of the lesser sac or tumor fixation could be inspected.

#### Greater omentum and bowel

Half of the studies specifically reported assessing greater omentum and small bowel. Retraction of the greater omentum toward the left upper quadrant and elevation of the transverse colon allows inspection of the root of mesenteries, duodenum, the proximal part of jejunum, and the ligament of Treitz. The surface of the small and large intestines was assessed in 15 studies^12,14,30,36,40,45,47,55,61,64,70,71,81,85,87^ (21%), while the inspection of the spleen’s surface and the hepatorenal recess was reported in 3 studies (4%)^14,40,88^.

#### Pelvic cavity

Examination of the pelvic cavity was reported in 27 (38%) studies by elevating the foot end of the table (Trendelenburg)^12,13,25,27,33,34,36,37,40,41,45,47,53,54,57,62,65,69,74,79,85,92,95,96,99,142^. Nine studies (12%) reported the evaluation of the ovaries and the fallopian tubes to rule out ovarium metastases^14,30,36,38,54,87,93,98,106^. Furthermore, inspection for peritoneal deposits of tumor cells in the Douglas pouch was reported explicitly in three studies (4%)^51,88,114^.

#### Esophageal hiatus

The evaluation of the esophageal hiatus was discussed in nine studies^34,38,39,44,61,81,83,86,89,95^. Primarily, for tumors of the gastroesophageal junction, exploration of the diaphragmatic hiatus was achieved through an incision and a blunt dissection to the phrenoesophageal peritoneal fold.

#### Classification of peritoneal dissemination

Three studies reported a classification system to assess the extent of peritoneal carcinomatosis^12,31,65^. The peritoneal cavity was evaluated either using the Peritoneal Cancer Index (PCI)^12,31^ or according to the Japanese Research Society for Gastric Cancer (P0,1,2,3)^65^.

#### Complications

Perioperative complications after staging laparoscopy in gastric cancer were reported in 13 studies^11,14,32,37,40,49,59,61,64,65,86,95,101^. The most common reported complication was intestinal perforation (30%), followed by myocardial infarction and blood loss (23%). Vascular injury, diaphragmatic perforation, and pulmonary infection were documented in two studies (15%), while ileum perforation, urine infection, and subcutaneous emphysema were reported least frequently (7%).

#### Intraoperative peritoneal lavage

Out of 29 studies assessing the timing of peritoneal washings during staging laparoscopy, 25 (86%) reported lavage at the beginning of the procedure and before manipulation of the primary tumor^12–14,25,26,28,30,32,34,36,44,47–51,59,61,63,65,78,83,84,93,95^. In contrast, four studies (14%) reported peritoneal lavage performed after the abdominal cavity inspection and/or peritoneal biopsy^3,70,79,80^. No comparative studies were identified about intraoperative peritoneal lavage timing during staging laparoscopy. In two retrospective studies from the East, cytology of the fluid was performed only in tumors with serosal invasion as a supplementary investigation^51,80^. Moreover, according to 21 studies, a sample for cytological examination was obtained in the presence of ascites^14,28,30,34,37–40,48,50,51,58,61,63,78,79,84,85,98^.

#### Intraperitoneal lavage aspiration

No studies reported the timing of intra-abdominal fluid injection and aspiration. Two studies reported a 3–5-min interval between peritoneal lavage and fluid aspiration^14,70^. Before the collection of the fluid, a gentle peritoneal agitation was performed, as reported in eight studies (27%).

#### The volume of fluid and aspiration

**Figure 3:**
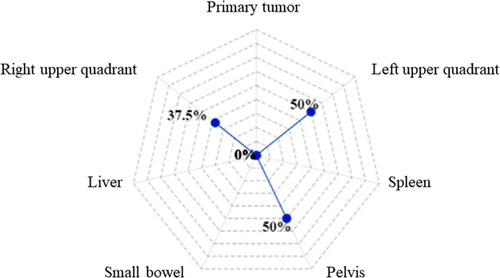
Radar chart evaluating the areas of peritoneal fluid aspiration during staging laparoscopy among included studies.

Detailed information on the volume of peritoneal lavage fluid was reported in 38 studies^11,12,14,19,25,26,28–34,36,37,41,42,44,46,47,49–51,54,57,59,61,63–65,70,73,78–80,83,84,95^. With a range of 20–1000 ml, the most common volume of intraperitoneal lavage in the included reports was 200 ml (8/38 studies, 19%). All peritoneal washings were conducted with 0.9% saline solution. The following regions were described as areas of fluid aspiration: right and left upper abdominal quadrants lesser sac, right paracolic sulcus or quadrant, left paracolic sulcus or quadrant, pouch of Douglas, pelvic floor, subhepatic space, hepatorenal recess, splenic recess, and over the primary tumor area. Reported regions of intraperitoneal lavage aspiration are depicted in Figure 3. Although no studies compared the fluid volume aspiration, the amount of fluid sent for cytological evaluation ranged from 50 to 100 ml, according to 12 reports^11,12,14,19,25,26,34,41,46,47,51,54,65,70,79,80,93^. Most authors did not routinely describe the storage conditions of the aspirated intraperitoneal fluid. However, two studies reported transportation in universal containers with no additives^79^ or in conical tubes^36^. A summary of surgical techniques of staging laparoscopy in gastric cancer followed by peritoneal lavage assessment is shown in Supplementary Material Table 3, Supplemental Digital Content 4, http://links.lww.com/JS9/A857.

## Discussion

Nearly four decades ago, Gross *et al*.^201^ reported in British Medical Journal that staging laparoscopy in gastric cancer patients was a clinically useful and safe procedure. Since that time, outcomes of over 5000 patients were reported in studies conducted in countries with the highest gastric cancer incidence and treatment experience. Advancements in minimally-invasive surgery have occurred since the time that the first documented peritoneal nodule biopsy was reported. Staging laparoscopy can now be enhanced with intraoperative ultrasonography and fluorescence imaging, which allow for staging optimization and treatment tailoring in cancer patients^202^. Our own group is currently evaluating the role of indocyanine-green (ICG) dye in nodal staging during diagnostic laparoscopy in a prospective and multi-institutional setting^203^. The current systematic review summarized the available data on staging laparoscopy technique and peritoneal fluid assessment among gastric cancer patients. Despite significant heterogeneity among the 212 included studies, several common characteristics of the procedure and cytological evaluation for gastric cancer were identified. For example, open Hasson was the method of choice to access the peritoneal cavity in 65% of studies, followed by establishing a pneumoperitoneum at 10–12 mmHg. Most frequently, the patient was positioned supine (70%), while a 30° scope and three ports were used to assess the peritoneal cavity clockwise (72%, 77%, and 85%, respectively) (Fig. 1). Right and left upper abdomen quadrants were the predominant area of laparoscopic exploration (both 65%), followed by primary tumor region (54%), liver and pelvis (both 30%), and small bowel and spleen (19% and 17%, respectively) (Fig. 2). Regions of peritoneal lavage and aspiration were limited to pelvis (50%), followed by right and left upper abdomen quadrants (37.5% and 50%, respectively) (Fig. 3). The technique of staging laparoscopy likely differs according to the surgeon’s preference, patient habitus, as well as primary tumor location. Although intraoperative findings regarding peritoneal carcinomatosis significantly differ among patients with gastric- and gastroesophageal junction cancers^39^, the proximal location of the tumor may require additional locoregional assessment, including dissection of phrenoesophageal ligament and hiatus. Meanwhile, cephalad extension of the disease should be additionally evaluated with endosonography, with possible fine-needle aspiration of suspected lymph nodes^204^. For tumors located in the posterior gastric wall, dissection of the omental bursa should be considered, despite an increased risk of postoperative complications^3,54^. Special attention should be paid to possible duodenum and hepatoduodenal ligament infiltration in distal gastric cancers. Additional evaluation of carcinoembryonic antigen (CEA) and carbohydrate antigen 19-9 (CA 19-9) may be considered in such clinical settings^205^. Despite significant improvements in imaging over the last two decades^206^, radiological assessment of small bowel and small bowel mesentery remains challenging^207^. The abdominal area is crucial to access for peritoneal carcinomatosis and may be a reason for increased false-negative rates at staging laparoscopy^41^. To examine the small bowel mesentery and transverse mesocolon, intraoperative port placement and patient positioning modifications may be required. In a non-standardized setting, such adjustments may be time-consuming, raising a question of cost-effectiveness. Staging laparoscopy provides financial benefits in certain case-based scenarios, including signet ring histology, poor tumor differentiation, and lymphadenopathy^208^. From a surgical perspective, nodal involvement is one of the critical prognostic factors in gastric cancer^209^. However, estimating the resectability of bulky nodal disease around the celiac axis and its tributaries (N2 trier) is limited. Concomitant with the increasing implementation of minimally-invasive surgery in gastric cancer, a higher median lymph node harvest during gastrectomy has been observed^210^. Since laparoscopic and robotic techniques allow maintenance of oncological radicality and low mortality rate^211^, it has been suggested that extensive nodal assessment during staging laparoscopy may be used only when distant nodal disease is suspected. Staging laparoscopy combined with peritoneal cytology status may impact therapeutic decision-making^212^. When peritoneal disease is detected, palliative systemic or a combination of systemic and intraperitoneal chemotherapy is often indicated^4^. Administration of chemotherapy may converse with the positive cytology status, resulting in improved disease-specific survival (DSS)^147^. However, recommendations for routine laparoscopic workups vary between guidelines. Initially, the procedure was indicated for cT3-T4 tumors only^213^. NCCN guidelines recommend performing staging laparoscopy for cT1b or higher^214^, while the Japanese Gastric Cancer Association (JGCA) restricts staging laparoscopy to advanced tumors with neoadjuvant chemotherapy indications^215^. Generally, staging laparoscopy is suggested in all potentially resectable gastric cancer patients since considered a safe and minimally-invasive procedure^216,217^. Although the complication rate is likely low, serious adverse events can occur, particularly during intraoperative biopsies nearby vulnerable structures^95^. This systematic review did not aim to assess the diagnostic accuracy of laparoscopy as such. Leake *et al*.^218^ analyzed indications for diagnostic laparoscopy prior to curative-intent resection of gastric cancer over a decade ago. The accuracy of staging laparoscopy was independently evaluated according to T, N, and M stages. The accuracy assessment varied between 67–92.9% for the primary tumor, 64.3–66.7% for lymph node involvement, and 85–100% for distant metastases, including liver and peritoneal dissemination. Despite significant heterogeneity among studies evaluating cytological assessment of peritoneal lavage in gastric cancer patients, its contribution to staging laparoscopy for treatment decision-making has been underlined^37^. Conventional cytological evaluation (Papanicolaou or hematoxylin and eosin stains) presented low sensitivity and a poor negative predictive value, which led to the development of advanced techniques and improvement in detecting free cancer cells – immunoassays, immunohistochemistry (IHC), and reverse RT-PCR^219^. The latter is the most common alternative method for peritoneal lavage evaluation^70,115,129,133,135,145,155,167,172,178–180,186,191,193,199^. Although the sensitivity and specificity of the cytological assessment with several biomarkers (CEA, Ca19.9, Ca72-4, Ca15-3, AFP, cytokeratin 19, CYFRA 21.1) are ambiguous, the accuracy of the analysis increases with the clinical stage, reaching up to 87% in pT4 tumors. Moreover, patients with peritoneal recurrence will more likely be identified by a combination of RT-PCR and the cytological assay^193^. Conversely, RT-PCR detection rates may be unproportionally high due to CEA-mRNA expression in non-tumor cells^105^. Detecting peritoneal disease or positive cytology has significant consequences for gastric cancer patients’ treatment, particularly in the multimodal therapy setting^5^.

Although lacking standardization, the technique of staging laparoscopy has undoubtedly evolved over the last decades. Implementing narrow band imaging (NBI)^220^, near-infrared (NIR) fluorescent and ICG technologies may increase the accuracy of both staging laparoscopy and more complex minimally-invasive gastric cancer procedures^221^. However, objective measures are required to support the true impact of modern technologies on cancer surgery^222^. Compared with other surgical and staging procedures which established consensus on ‘how to do it’^223,224^, staging laparoscopy and peritoneal fluid assessment in gastric cancer patients remain highly variable. Although the most common approach to the procedure was pointed out in the current study, the best approach cannot be recommended due to the lack of studies directly comparing technical aspects intraoperatively. New research insights and initiatives to reach consensus globally are warranted. This systematic review has several limitations. Due to the use of various techniques for staging laparoscopy and peritoneal fluid assessment, the pooling of data was impossible. Moreover, selective outcome reporting in included studies might have led to clinical heterogeneity, and thus, our results should be interpreted cautiously. In conclusion, this systematic review demonstrated a high heterogeneity in the technique of staging laparoscopy and peritoneal fluid assessment in gastric cancer patients. Further research and initiatives to reach a consensus on the standardization of the procedure are warranted.

## Ethical approval

Not applicable.

## Consent

Not applicable.

## Sources of funding

None, however the First author is a scholar of the Polish National Agency for Academic Exchange (NAWA) Franciszek Walczak program, which allowed conducting this study as a Research Fellow at the Department of Surgery, Ohio State University, Wexner Medical Center, Columbus, Ohio, USA.

## Author contribution

K.R.-P.: conceptualization, search strategy, data retrieval, and writing – original manuscript, editing, and reviewing; M.E.: search strategy, data retrieval, and writing – original manuscript, editing, and reviewing; Z.P. and K.S.: data retrieval and writing – original manuscript, editing, and reviewing; W.P., T.M.P., and B.P.L.W.: correction and finalization of the manuscript and reviewing discussion.

## Conflicts of interest disclosure

There are no conflicts of interest.

## Research registration unique identifying number (UIN)

The protocol for this systematic review was registered on PROSPERO database (CRD: 42022306746).

## Guarantor

Karol Rawicz-Pruszyński.

## Data availability statement

Supplementary tables summarize the study search strategy and result in retrieval comprehensively. The authors confirm that the data supporting the findings of this study are available within the article and its supplementary materials, Supplemental Digital Content 4, http://links.lww.com/JS9/A857.

## Provenance and peer review

Not commissioned, externally peer-reviewed.

## Supplementary Material
