# Supplementary material for: Techniques of staging laparoscopy and peritoneal fluid assessment in gastric cancer: a systematic review
Source: Int J Surg. 2023 Aug 14;109(11):3578–89. doi: 10.1097/JS9.0000000000000632 (PMC10651295; doi:10.1097/JS9.0000000000000632)
Supplement: Supplementary file 1 [file js9-109-3578-s001.docx]

**
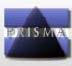
PRISMA 2020 Checklist**

|  |  |  |  |  |  |  |  |  |  |  |  |  |
| --- | --- | --- | --- | --- | --- | --- | --- | --- | --- | --- | --- | --- |
|  | **Section and** |  |  | **Item** |  |  |  |  |  | **Location** |  |  |
|  |  |  |  |  |  |  | **Checklist item** |  |  | **where item** |  |  |
|  | **Topic** |  |  | **#** |  |  |  |  |  |  |  |  |
|  |  |  |  |  |  |  |  |  |  | **is reported** |  |  |
|  |  |  |  |  |  |  |  |  |  |  |  |  |
|  |  | |  |  |  |  |  |  |  |  |  |  |
|  | **TITLE** | |  |  |  |  |  |  |  |  |  |  |
|  | Title | | 1 | |  |  | Identify the report as a systematic review. | |  | Page 1 |  |  |
|  |  | |  |  |  |  |  |  |  |  |  |  |
|  |  | |  |  |  |  |  |  |  |  |  |  |
|  | **ABSTRACT** | |  |  |  |  |  |  |  |  |  |  |
|  | Abstract | | 2 | |  |  | See the PRISMA 2020 for Abstracts checklist. | |  | Page 2 |  |  |
|  |  | |  |  |  |  |  |  |  |  |  |  |
|  |  | |  |  |  |  |  |  |  |  |  |  |
|  | **INTRODUCTION** | |  |  |  |  |  |  |  |  |  |  |
|  | Rationale | | 3 | |  |  | Describe the rationale for the review in the context of existing knowledge. | |  | Page 3-4 |  |  |
|  |  | |  | |  |  |  | |  |  |  |  |
|  | Objectives | | 4 | |  |  | Provide an explicit statement of the objective(s) or question(s) the review addresses. | |  | Page 4 |  |  |
|  |  | |  |  |  |  |  |  |  |  |  |  |
|  |  | |  |  |  |  |  |  |  |  |  |  |
|  | **METHODS** | |  |  |  |  |  |  |  |  |  |  |
|  | Eligibility criteria | | 5 | |  |  | Specify the inclusion and exclusion criteria for the review and how studies were grouped for the syntheses. | |  | Page 5-6 |  |  |
|  |  | |  | |  |  |  | |  |  |  |  |
|  | Information | | 6 | |  |  | Specify all databases, registers, websites, organisations, reference lists and other sources searched or consulted to identify studies. Specify the | |  | Page 5-6 |  |  |
|  | sources | |  |  |  |  | date when each source was last searched or consulted. | |  |  |  |  |
|  |  | |  | |  |  |  | |  |  |  |  |
|  | Search strategy | | 7 | |  |  | Present the full search strategies for all databases, registers and websites, including any filters and limits used. | |  | Page 5-6 |  |  |
|  |  | |  | |  |  |  | |  |  |  |  |
|  | Selection process | | 8 | |  |  | Specify the methods used to decide whether a study met the inclusion criteria of the review, including how many reviewers screened each record | |  | Page 5-6 |  |  |
|  |  |  |  |  |  |  | and each report retrieved, whether they worked independently, and if applicable, details of automation tools used in the process. | |  |  |  |  |
|  |  | |  | |  |  |  | |  |  |  |  |
|  | Data collection | | 9 | |  |  | Specify the methods used to collect data from reports, including how many reviewers collected data from each report, whether they worked | |  | Page 5-6 |  |  |
|  | process | |  |  |  |  | independently, any processes for obtaining or confirming data from study investigators, and if applicable, details of automation tools used in the | |  |  |  |  |
|  |  |  |  |  |  |  | process. | |  |  |  |  |
|  |  | |  |  | |  |  | |  |  |  |  |
|  | Data items | |  | 10a | |  | List and define all outcomes for which data were sought. Specify whether all results that were compatible with each outcome domain in each | |  | Page 5-6 |  |  |
|  |  |  |  |  |  |  | study were sought (e.g. for all measures, time points, analyses), and if not, the methods used to decide which results to collect. | |  |  |  |  |
|  |  |  |  |  | |  |  | |  |  |  |  |
|  |  |  |  | 10b | |  | List and define all other variables for which data were sought (e.g. participant and intervention characteristics, funding sources). Describe any | |  | Page 5-6 |  |  |
|  |  |  |  |  |  |  | assumptions made about any missing or unclear information. | |  |  |  |  |
|  |  | |  | | |  |  | |  |  |  |  |
|  | Study risk of bias | | 11 | | |  | Specify the methods used to assess risk of bias in the included studies, including details of the tool(s) used, how many reviewers assessed each | |  | Page 5-6 |  |  |
|  | assessment | |  |  |  |  | study and whether they worked independently, and if applicable, details of automation tools used in the process. | |  |  |  |  |
|  |  | |  | | |  |  | |  |  |  |  |
|  | Effect measures | | 12 | | |  | Specify for each outcome the effect measure(s) (e.g. risk ratio, mean difference) used in the synthesis or presentation of results. | |  | Page 5-6 |  |  |
|  |  | |  |  | |  |  | |  |  |  |  |
|  | Synthesis | |  | 13a | |  | Describe the processes used to decide which studies were eligible for each synthesis (e.g. tabulating the study intervention characteristics and | |  | Page 5-6 |  |  |
|  | methods | |  |  |  |  | comparing against the planned groups for each synthesis (item #5)). | |  |  |  |  |
|  |  |  |  |  | |  |  | |  |  |  |  |
|  |  |  |  | 13b | |  | Describe any methods required to prepare the data for presentation or synthesis, such as handling of missing summary statistics, or data | |  | Page 5-6 |  |  |
|  |  |  |  |  |  |  | conversions. | |  |  |  |  |
|  |  |  |  |  | |  |  | |  |  |  |  |
|  |  |  |  | 13c | |  | Describe any methods used to tabulate or visually display results of individual studies and syntheses. | |  | Page 5-6 |  |  |
|  |  |  |  |  | |  |  | |  |  |  |  |
|  |  |  |  | 13d | |  | Describe any methods used to synthesize results and provide a rationale for the choice(s). If meta-analysis was performed, describe the | |  | Page 5-6 |  |  |
|  |  |  |  |  |  |  | model(s), method(s) to identify the presence and extent of statistical heterogeneity, and software package(s) used. | |  |  |  |  |
|  |  |  |  |  | |  |  | |  |  |  |  |
|  |  |  |  | 13e | |  | Describe any methods used to explore possible causes of heterogeneity among study results (e.g. subgroup analysis, meta-regression). | |  | Page 5-6 |  |  |
|  |  |  |  |  | |  |  | |  |  |  |  |
|  |  |  |  | 13f | |  | Describe any sensitivity analyses conducted to assess robustness of the synthesized results. | |  | Page 5-6 |  |  |
|  |  | |  | | |  |  | |  |  |  |  |
|  | Reporting bias | | 14 | | |  | Describe any methods used to assess risk of bias due to missing results in a synthesis (arising from reporting biases). | |  | Page 5-6 |  |  |
|  | assessment | |  |  |  |  |  |  |  |  |  |  |
|  |  | |  | | |  |  | |  |  |  |  |
|  | Certainty | | 15 | | |  | Describe any methods used to assess certainty (or confidence) in the body of evidence for an outcome. | |  | Page 5-6 |  |  |
|  | assessment | |  |  |  |  |  |  |  |  |  |  |
|  |  |  |  |  |  |  |  |  |  |  |  |  |

**
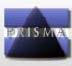
PRISMA 2020 Checklist**

|  |  |  |  |  |  |  |  |  |  |  |  |  |
| --- | --- | --- | --- | --- | --- | --- | --- | --- | --- | --- | --- | --- |
|  | **Section and** |  |  | **Item** |  |  |  |  |  | **Location** |  |  |
|  |  |  |  |  |  |  | **Checklist item** |  |  | **where item** |  |  |
|  | **Topic** |  |  | **#** |  |  |  |  |  |  |  |  |
|  |  |  |  |  |  |  |  |  |  | **is reported** |  |  |
|  |  |  |  |  |  |  |  |  |  |  |  |  |
|  |  | |  |  |  |  |  |  |  |  |  |  |
|  | **RESULTS** | |  |  |  |  |  |  |  |  |  |  |
|  | Study selection | |  | 16a | |  | Describe the results of the search and selection process, from the number of records identified in the search to the number of studies included in | |  | Page 6-12 |  |  |
|  |  |  |  |  |  |  | the review, ideally using a flow diagram. | |  |  |  |  |
|  |  |  |  |  | |  |  | |  |  |  |  |
|  |  |  |  | 16b | |  | Cite studies that might appear to meet the inclusion criteria, but which were excluded, and explain why they were excluded. | |  | Page 6-12 |  |  |
|  |  | |  | | |  |  | |  |  |  |  |
|  | Study | | 17 | | |  | Cite each included study and present its characteristics. | |  | Page, 6-12 |  |  |
|  | characteristics | |  |  |  |  |  |  |  |  |  |  |
|  |  | |  | | |  |  | |  |  |  |  |
|  | Risk of bias in | | 18 | | |  | Present assessments of risk of bias for each included study. | |  | Page 6-12 |  |  |
|  | studies | |  |  |  |  |  |  |  |  |  |  |
|  |  | |  | | |  |  | |  |  |  |  |
|  | Results of | | 19 | | |  | For all outcomes, present, for each study: (a) summary statistics for each group (where appropriate) and (b) an effect estimate and its precision | |  | Page 6-12 |  |  |
|  | individual studies | |  |  |  |  | (e.g. confidence/credible interval), ideally using structured tables or plots. | |  |  |  |  |
|  |  | |  |  | |  |  | |  |  |  |  |
|  | Results of | |  | 20a | |  | For each synthesis, briefly summarise the characteristics and risk of bias among contributing studies. | |  | Page 6-12 |  |  |
|  | syntheses | |  |  |  |  |  |  |  |  |  |  |
|  |  |  |  | 20b | |  | Present results of all statistical syntheses conducted. If meta-analysis was done, present for each the summary estimate and its precision (e.g. | |  |  |  |  |
|  |  |  |  |  |  |  |  |  |  | Page |  |  |
|  |  |  |  |  |  |  | confidence/credible interval) and measures of statistical heterogeneity. If comparing groups, describe the direction of the effect. | |  | 6-12 |  |  |
|  |  |  |  |  | |  |  | |  |  |  |  |
|  |  |  |  | 20c | |  | Present results of all investigations of possible causes of heterogeneity among study results. | |  | Page 6-12 |  |  |
|  |  |  |  |  | |  |  | |  |  |  |  |
|  |  |  |  | 20d | |  | Present results of all sensitivity analyses conducted to assess the robustness of the synthesized results. | |  | Page 6-12 |  |  |
|  |  | |  | | |  |  | |  |  |  |  |
|  | Reporting biases | | 21 | | |  | Present assessments of risk of bias due to missing results (arising from reporting biases) for each synthesis assessed. | |  | Page 6-12 |  |  |
|  |  | |  | | |  |  | |  |  |  |  |
|  | Certainty of | | 22 | | |  | Present assessments of certainty (or confidence) in the body of evidence for each outcome assessed. | |  | Page 6-12 |  |  |
|  | evidence | |  |  |  |  |  |  |  |  |  |  |
|  |  | |  |  |  |  |  |  |  |  |  |  |
|  |  | |  |  |  |  |  |  |  |  |  |  |
|  | **DISCUSSION** | |  |  |  |  |  |  |  |  |  |  |
|  | Discussion | |  | 23a | |  | Provide a general interpretation of the results in the context of other evidence. | |  | Page 13-16 |  |  |
|  |  |  |  |  | |  |  | |  |  |  |  |
|  |  |  |  | 23b | |  | Discuss any limitations of the evidence included in the review. | |  | Page 13-16 |  |  |
|  |  |  |  |  | |  |  | |  |  |  |  |
|  |  |  |  | 23c | |  | Discuss any limitations of the review processes used. | |  | Page 13-16 |  |  |
|  |  |  |  |  | |  |  | |  |  |  |  |
|  |  |  |  | 23d | |  | Discuss implications of the results for practice, policy, and future research. | |  | Page 13-16 |  |  |
|  |  | |  | | |  |  |  |  |  |  |  |
|  |  | | | | |  |  |  |  |  |  |  |
|  | **OTHER INFORMATION** | | | | |  |  |  |  |  |  |  |
|  | Registration and | |  | 24a | |  | Provide registration information for the review, including register name and registration number, or state that the review was not registered. | |  | Page 5-6 |  |  |
|  | protocol | |  | 24b | |  | Indicate where the review protocol can be accessed, or state that a protocol was not prepared. | |  |  |  |  |
|  |  |  |  |  |  |  |  |  |  | Page 5-6 |  |  |
|  |  |  |  |  | |  |  | |  |  |  |  |
|  |  |  |  | 24c | |  | Describe and explain any amendments to information provided at registration or in the protocol. | |  | Page 5-6 |  |  |
|  |  | |  | | |  |  | |  |  |  |  |
|  | Support | | 25 | | |  | Describe sources of financial or non-financial support for the review, and the role of the funders or sponsors in the review. | |  | Page 17 |  |  |
|  |  | |  | | |  |  | |  |  |  |  |
|  | Competing | | 26 | | |  | Declare any competing interests of review authors. | |  | Page 17 |  |  |
|  | interests | |  |  |  |  |  |  |  |  |  |  |
|  |  | |  | | |  |  | |  |  |  |  |
|  | Availability of | | 27 | | |  | Report which of the following are publicly available and where they can be found: template data collection forms; data extracted from included | |  | Page 4-5 |  |  |
|  | data, code and | |  |  |  |  | studies; data used for all analyses; analytic code; any other materials used in the review. | |  |  |  |  |
|  | other materials | |  |  |  |  |  |  |  |  |  |  |
|  |  |  |  |  |  |  |  |  |  |  |  |  |
|  |  |  |  |  |  |  |  |  |  |  |  |  |

*From:* Page MJ, McKenzie JE, Bossuyt PM, Boutron I, Hoffmann TC, Mulrow CD, et al. The PRISMA 2020 statement: an updated guideline for reporting systematic reviews. BMJ 2021;372:n71. doi:

10.1136/bmj.n71

For more information, visit: <http://www.prisma-statement.org/>
