# Supplementary material for: Techniques of staging laparoscopy and peritoneal fluid assessment in gastric cancer: a systematic review
Source: Int J Surg. 2023 Aug 14;109(11):3578–89. doi: 10.1097/JS9.0000000000000632 (PMC10651295; doi:10.1097/JS9.0000000000000632)
Supplement: Supplementary file 2 [file js9-109-3578-s002.docx]

**Figure.1** PRISMA flowchart for staging laparoscopy technique.

Records after duplicates removed *before screening*:

(n=500)

Records identified through database searching:

(n= 1632)

**Identification**

Records excluded:

(n =1003 )

- Studies not about diagnostic laparoscopy (n=990)
- Reviews, editorials (n=9)
- Case reports, conferences (n=2)
- Studies published in a language other than English (n=1)
- Animal Models (n=1)

Records screened

(n =1132 )

**Screening**

Full Text Reports not retrieved

(n =29 )

Reports sought for retrieval

(n = 129 )

Reports excluded:

(n=16)

- Studies without description of the diagnostic laparoscopy procedure (n=16)

Reports assessed for eligibility

(n =100 )

Studies included in review

(n = 84)

**Included**
