# Supplementary material for: Techniques of staging laparoscopy and peritoneal fluid assessment in gastric cancer: a systematic review
Source: Int J Surg. 2023 Aug 14;109(11):3578–89. doi: 10.1097/JS9.0000000000000632 (PMC10651295; doi:10.1097/JS9.0000000000000632)
Supplement: Supplementary file 3 [file js9-109-3578-s003.docx]

**Figure.2** PRISMA flowchart for peritoneal fluid assessment.

Records after duplicates removed *before screening*

(n=1019)

Records identified through database searching:

(n = 2190 )

**Identification**

Records excluded:

(n = 852)

- Studies not on assessment of peritoneal lavage fluid ( n=771)
- Systematic reviews, meta-analysis, reviews, editorials/letters (n=23)
- Case reports, posters, conferences (n=43)
- Studies published in a language other than English (n=2)
- Animal models (n=13)

Records screened

(n =1171 )

**Screening**

Records sought for retrieval

(n = 319)

Full Text Records not retrieved

(n =189 )

Records assessed for eligibility

(n = 130)

Records excluded:

(n=2)

- Duplicates (n = 2 )

Records included in review

(n = 128 )

**Included**
