# Supplementary material for: Techniques of staging laparoscopy and peritoneal fluid assessment in gastric cancer: a systematic review
Source: Int J Surg. 2023 Aug 14;109(11):3578–89. doi: 10.1097/JS9.0000000000000632 (PMC10651295; doi:10.1097/JS9.0000000000000632)
Supplement: Supplementary file 4 [file js9-109-3578-s004.docx]

**Techniques of staging laparoscopy and peritoneal fluid assessment in gastric cancer: a systematic review.**

**Supplementary Material Tables**

**Supplementary Material Table 1.** Search strategy for staging laparoscopy technique.

| Database searched  (September 16, 2021) | Years of coverage | Search terms | Records | Records after duplicates removed |
| --- | --- | --- | --- | --- |
| Embase | 1971 - Present | ('stomach tumor'/mj/exp OR 'gastroesophageal cancer'/mj/de OR 'gastroesophageal carcinoma'/mj/de OR 'esophagogastric cancer'/mj/de OR (((stomach OR gastric* OR gastroesophag* OR gastro-esophag* OR gastro-oesophag* OR esophagogastr* OR oesophagogastr* OR cardia) NEAR/6 (cancer* OR neoplas* OR tumo* OR carcinoma* OR adenocarcinoma* OR malign*))):ti) AND (laparoscopy/mj/de OR laparoscope/mj/de OR (laparoscop* OR peritoneoscop*):ti) AND ('cancer staging'/exp OR 'diagnostic procedure'/de OR diagnosis/de OR 'stomach tumor'/exp/dm_di OR 'diagnostic value'/de OR 'diagnostic accuracy'/de OR 'diagnostic test accuracy study'/de OR 'cancer diagnosis'/exp OR 'diagnostic peritoneal lavage'/de OR 'diagnostic test'/de OR 'early diagnosis'/de OR 'differential diagnosis'/de OR 'diagnostic error'/de OR (staging OR diagnos*):ab,ti) NOT ([animals]/lim NOT [humans]/lim) NOT ([conference abstract]/lim OR [note]/lim OR [letter]/lim OR [editorial]/lim) AND [english]/lim NOT ('systematic review'/de OR 'meta analysis'/de OR ((systematic* NEAR/3 review*) OR (meta-analy*)):ti) | 957 | 481 |
| Medline ALL | 1946 - Present | (mh Stomach Neoplasms / OR (((stomach OR gastric* OR gastroesophag* OR gastro-esophag* OR gastro-oesophag* OR esophagogastr* OR oesophagogastr* OR cardia) ADJ6 (cancer* OR neoplas* OR tumo* OR carcinoma* OR adenocarcinoma* OR malign*))).ti.) AND (mh laparoscopy/ OR mh laparoscopes/ OR (laparoscop* OR peritoneoscop*).ti.) AND (exp Neoplasm Staging / OR Diagnostic Test, Routine / OR Diagnosis/ OR Early Detection of Cancer/ OR Peritoneal lavage/ OR Early diagnosis/ OR Diagnosis, differential / OR Diagnostic Errors/ OR (staging OR detection* OR diagnos*).ab,ti.) NOT (exp animals/ NOT humans/) NOT (conference abstract OR note OR letter OR editorial) AND english.la. NOT (systematic review/ OR meta analysis/ OR ((systematic* ADJ3 review*) OR (meta-analy*)).ti.) | 609 | 607 |
| Cochrane Central Register of Controlled Trials | 1992 - Present | ((((stomach OR gastric* OR gastroesophag* OR gastro-esophag* OR gastro-oesophag* OR esophagogastr* OR oesophagogastr* OR cardia) NEAR/6 (cancer* OR neoplas* OR tumo* OR carcinoma* OR adenocarcinoma* OR malign*))):ti) AND ((laparoscop* OR peritoneoscop*):ti) AND ((staging OR diagnos*):ab,ti) NOT (conference abstract OR note OR letter OR editorial NOT systematic review OR meta analysis OR ((systematic* NEAR/3 review*) OR (meta-analy*)):pt) | 66 | 44 |

**Supplementary Material Table 2.** Search strategy for peritoneal fluid assessment.

| Database searched  (September 16, 2021) | Years of coverage | Search Terms | Records | Records after duplicates removed |
| --- | --- | --- | --- | --- |
| Embase | 1971 - Present | ('stomach tumor'/exp OR 'gastroesophageal cancer'/de OR 'gastroesophageal carcinoma'/de OR 'esophagogastric cancer'/de OR (((stomach OR gastric* OR gastroesophag* OR gastro-esophag* OR gastro-oesophag* OR esophagogastr* OR oesophagogastr* OR cardia) NEAR/6 (cancer* OR neoplas* OR tumo* OR carcinoma* OR adenocarcinoma* OR malign*))):ab,ti) AND ('lavage fluid'/de OR 'peritoneum lavage'/de OR 'ascites fluid'/de OR 'ascites fluid cytology'/de OR **'stomach lavage'/de OR lavage/de OR ascites/de OR 'peritoneal fluid'/de OR 'malignant ascites'/de** OR ((lavage NEAR/3 (fluid* OR sample* OR specimen* OR Peritoneal* OR cytolog*)) OR (periton* NEAR/3 (wash* OR cytolog* **OR fluid***)) OR ascites):ab,ti) AND (cytology/exp OR microscopy/de OR 'polymerase chain reaction'/exp OR (**immunocytolog*** OR cytolog* OR cytodiagnos* OR assessment* OR microscop* OR (polymerase NEAR/3 chain NEAR/3 reaction*) OR Pcr):ab,ti) NOT ([animals]/lim NOT [humans]/lim) NOT ([conference abstract]/lim OR [note]/lim OR [letter]/lim OR [editorial]/lim) AND [english]/lim NOT ('systematic review'/de OR 'meta analysis'/de OR ((systematic* NEAR/3 review*) OR (meta-analy*)):ti) | 771 | 767 |
| Medline ALL | 1946 - Present | (exp Stomach Neoplasms/ OR (((stomach OR gastric* OR gastroesophag* OR gastro-esophag* OR gastro-oesophag* OR esophagogastr* OR oesophagogastr* OR cardia) ADJ6 (cancer* OR neoplas* OR tumo* OR carcinoma* OR adenocarcinoma* OR malign*))).ab,ti.) AND (Peritoneal Lavage / OR Ascites / OR Gastric Lavage / OR Ascitic Fluid / OR ((lavage ADJ3 (fluid* OR sample* OR specimen* OR Peritoneal* OR cytolog*)) OR (periton* ADJ3 (wash* OR cytolog* OR fluid*)) OR ascites).ab,ti.) AND (exp Cell Biology / OR Microscopy / OR exp Polymerase Chain Reaction / OR (immunocytolog* OR cytolog* OR cytodiagnos* OR assessment* OR microscop* OR (polymerase ADJ3 chain ADJ3 reaction*) OR Pcr).ab,ti.) NOT (exp animals/ NOT humans/) NOT (conference abstract/ OR note/ OR letter/ OR editorial/) AND (english).lg NOT (systematic review/ OR meta analysis/ OR ((systematic* ADJ3 review*) OR (meta-analy*)).ti.) | 592 | 67 |
| Web of Science Core Collection* | 1975 - Present | TS=(((((stomach OR gastric* OR gastroesophag* OR gastro-esophag* OR gastro-oesophag* OR esophagogastr* OR oesophagogastr* OR cardia) NEAR/5 (cancer* OR neoplas* OR tumo* OR carcinoma* OR adenocarcinoma* OR malign*)))) AND (((lavage NEAR/2 (fluid* OR sample* OR specimen* OR Peritoneal* OR cytolog*)) OR (periton* NEAR/2 (wash* OR cytolog* OR fluid*)) OR ascites)) AND ((immunocytolog* OR cytolog* OR cytodiagnos* OR assessment* OR microscop* OR (polymerase NEAR/2 chain NEAR/2 reaction*) OR Pcr))) | 731 | 258 |
| Cochrane Central Register of Controlled Trials | 1992 - Present | ((((stomach OR gastric* OR gastroesophag* OR gastro NEXT esophag* OR gastro NEXT oesophag* OR esophagogastr* OR oesophagogastr* OR cardia) NEAR/6 (cancer* OR neoplas* OR tumo* OR carcinoma* OR adenocarcinoma* OR malign*))):ab,ti) AND (((lavage NEAR/3 (fluid* OR sample* OR specimen* OR Peritoneal* OR cytolog*)) OR (periton* NEAR/3 (wash* OR cytolog* OR fluid*)) OR ascites):ab,ti) AND ((immunocytolog* OR cytolog* OR cytodiagnos* OR assessment* OR microscop* OR (polymerase NEAR/3 chain NEAR/3 reaction*) OR Pcr):ab,ti) | 96 | 79 |

**Supplementary Material Table 3.** A summary of surgical techniques of staging laparoscopy in gastric cancer followed by peritoneal lavage assessment^. 2,11,12,14,15,20,26-224,^

| **Variable** | **Technique / Outcome** | **Most used** |
| --- | --- | --- |
| Abdominal access | open Hasson; Verres needle | Open Hasson |
| Pneumoperitoneum | 8-15mm Hg | 10-12mm Hg |
| Number of Ports/Trocars | 2-5 | 3 |
| Position | RUQ, LUQ, (peri)umbilical right flank, left flank | RUQ, LUQ, (peri)umbilical |
| Size | 5-12mm | 5mm, 10mm, 12mm |
| Scope | 0-30° | 30° |
| Positioning | Supine, French, lithotomy | Supine |
| Orientation of exploration | Clockwise, Anti-clockwise | Clockwise |
| Regions of exploration | stomach (primary tumor) local tumor ingrowth, peritoneal surface, greater omentum, liver, Treitz ligament, duodenum, pancreas, celiac trunk, spleen, hilum of the liver, hepatoduodenal ligament, foramen of Winslow, RUQ, LUQ , small bowel, | Primary tumor, RUQ, LUQ, Liver, Small bowel, Pelvis, Spleen |
| Classification of peritoneal dissemination | PCI, Japanese | PCI |
| Complications | Pulmonary infection, diaphragmatic perforation, myocardial infarction, blood loss, intestinal perforation, pneumothorax, vascular injury | Intestinal perforation |
| Timing of peritoneal lavage | Beginning of the procedure, end of the procedure | Beginning of the procedure |
| Intraoperative presence of ascites | Sample for cytological examination | Sample for cytological examination |
| Time between lavage and fluid aspiration | 3-5 min | 3min |
| Volume of fluid aspiration | 50-1000ml | 200ml |
| Volume of fluid sent for cytology | 30-400ml | 100ml |

RUQ – right upper quadrant, LUQ – left upper quadrant, PCI – peritoneal cancer index
