# Supplementary material for: Techniques of staging laparoscopy and peritoneal fluid assessment in gastric cancer: a systematic review
Source: Int J Surg. 2023 Aug 14;109(11):3578–89. doi: 10.1097/JS9.0000000000000632 (PMC10651295; doi:10.1097/JS9.0000000000000632)

**Supplementary Figure 1**. PRISMA flowchart for staging laparoscopy techniques in gastric cancer patients


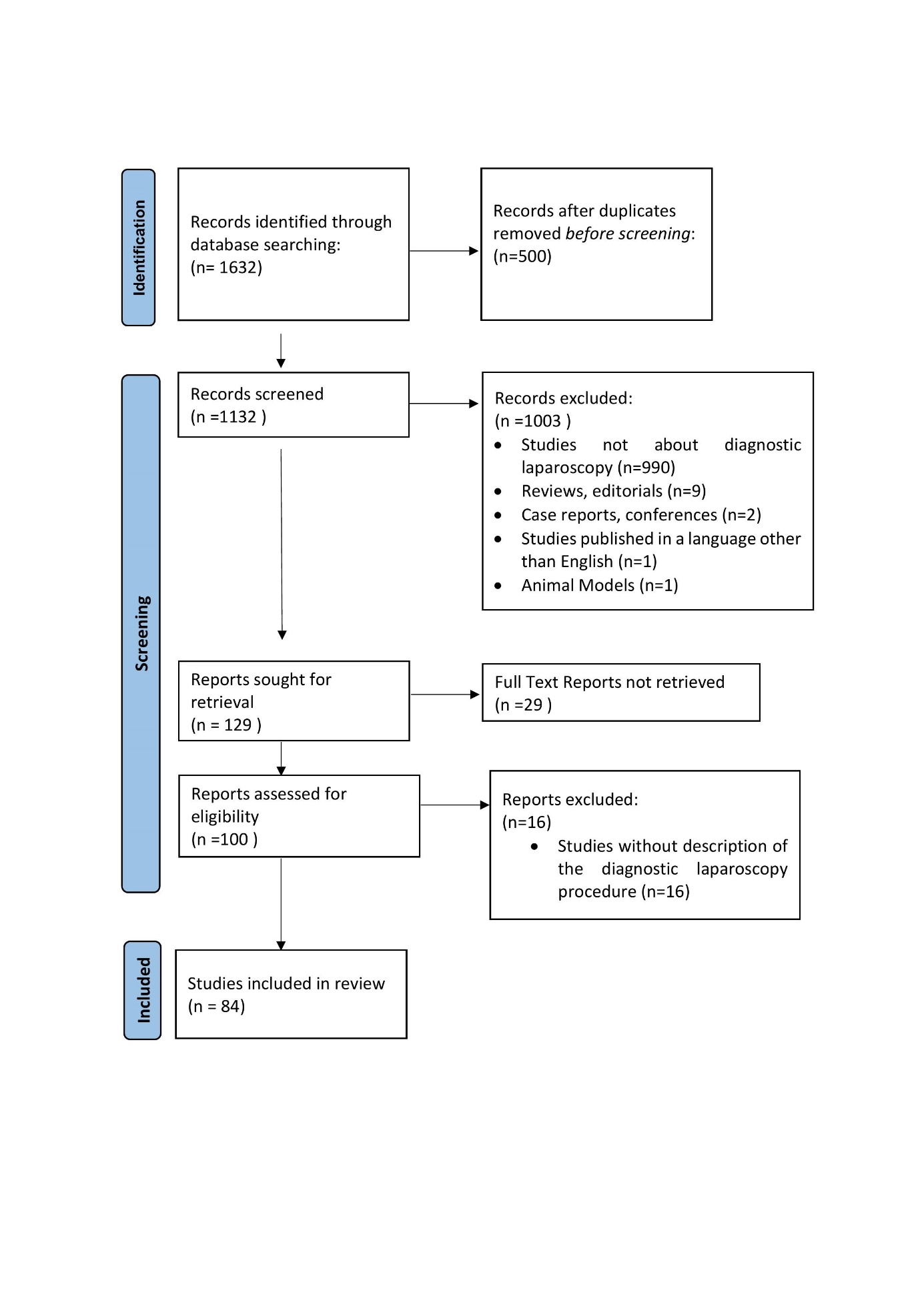


**Supplementary Figure 2**. PRISMA flowchart for peritoneal lavage assessment in gastric cancer patients


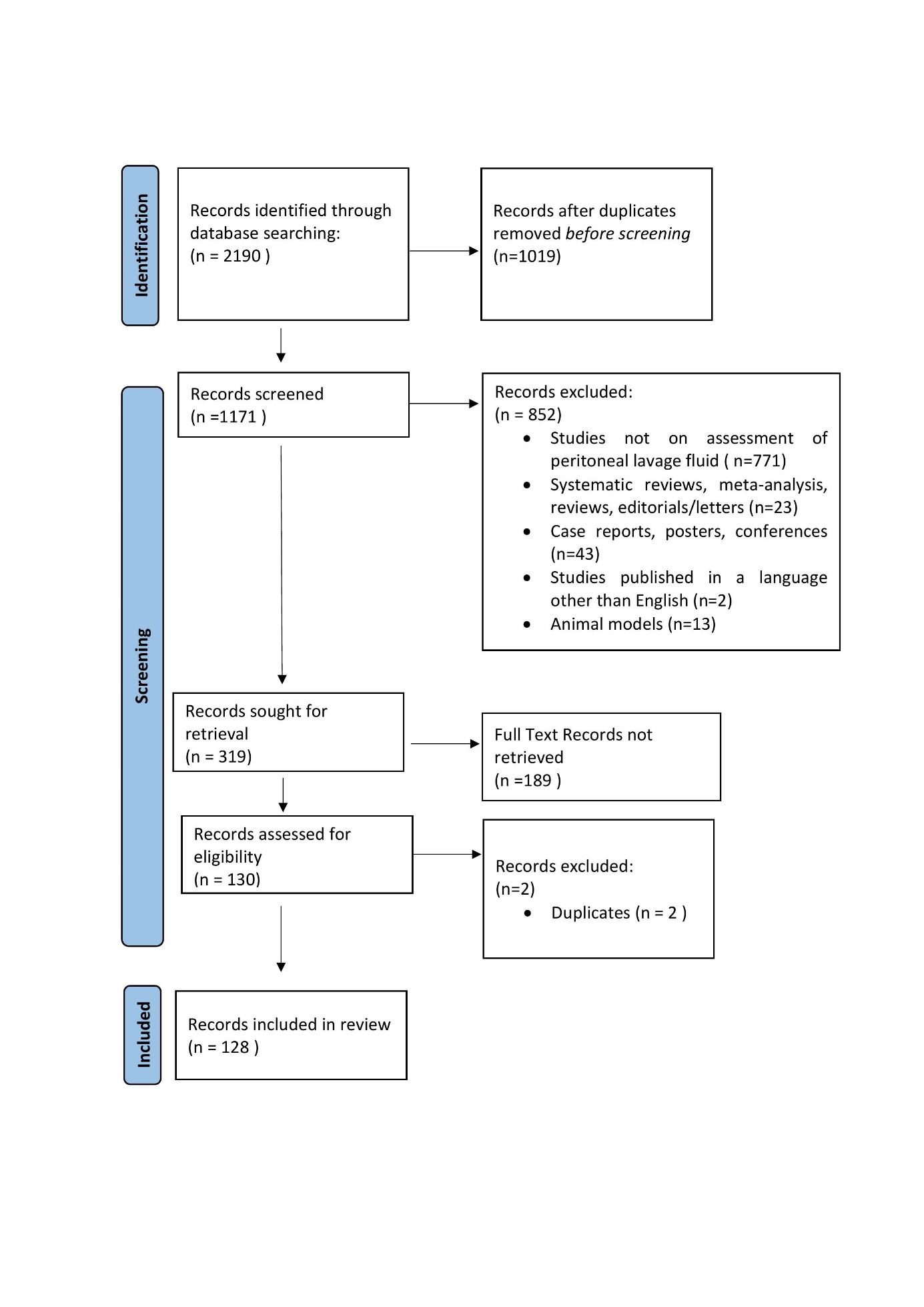

Supplement: Supplementary file 5 [file js9-109-3578-s005.docx]
